# Caring for Australians and New Zealanders with kidney Impairment guidelines commentary on the Kidney Disease: Improving Global Outcomes clinical practice guideline for management of diabetes and chronic kidney disease

**DOI:** 10.1111/imj.70317

**Published:** 2026-02-02

**Authors:** Hannah Wallace, Mia E. Abdy, Kathie Anderson, Effie Johns, Thu Nguyen, Carla Scuderi, Vincent Lee, David J. Tunnicliffe, Min Jun, on behalf CARI Guidelines Steering Committee, Emily See, Leanne Brown, Helen Coolican, Jonathan Craig, Vanessa Cullen, Jeffrey Ha, Rathika Krishnasamy, Kelly Lambert, Casey Light, Emmy O'Neill, Hannah Smith, Andrea Viecelli

**Affiliations:** ^1^ The George Institute for Global Health UNSW Sydney Sydney New South Wales Australia; ^2^ Western Health Chronic Disease Alliance Western Health Melbourne Victoria Australia; ^3^ Faculty of Medicine and Health University of Melbourne Melbourne Victoria Australia; ^4^ Sydney School of Public Health The University of Sydney Sydney New South Wales Australia; ^5^ Centre for Kidney Research The Children's Hospital at Westmead Sydney New South Wales Australia; ^6^ The Children's Hospital at Westmead Consumer Partner Melbourne Victoria Australia; ^7^ Department of Renal Medicine Te Toka Tumai Auckland Auckland New Zealand; ^8^ School of Pharmacy The University of Queensland Brisbane Queensland Australia; ^9^ Westmead Applied Research Centre The University of Sydney Sydney New South Wales Australia; ^10^ Faculty of Medicine and Health UNSW Sydney New South Wales Australia

**Keywords:** chronic kidney disease, diabetes mellitus, guidelines, shared decision‐making

## Abstract

Diabetes is a leading cause of kidney failure, and individuals with both diabetes and chronic kidney disease (CKD) experience significantly higher rates of complications and mortality. The international guideline developer Kidney Disease: Improving Global Outcomes (KDIGO) has produced clinical practice guidelines that reflect recent advances in pharmacotherapy for this population, extending beyond glycaemic control to include cardio‐renal benefits. However, these guidelines were developed without specific consideration of the healthcare systems, access issues and population needs in Australia and New Zealand. In response, the Caring for Australians and New Zealanders with Kidney Impairment (CARI) Guidelines Working Group has provided a regional commentary on the KDIGO 2022 guideline. This commentary highlights key recommendations and contextualises their implementation within the Australian and New Zealand healthcare environments. It addresses issues such as medication access, equity for Indigenous populations and the importance of shared decision‐making, aiming to support clinicians in delivering evidence‐based, locally relevant care for people living with diabetes and CKD.

## Introduction

Diabetes is the leading cause of kidney failure,^1^, ^2^ and patients with comorbid diabetes and chronic kidney disease (CKD) are at high risk of cardiovascular complications and mortality.^3^ There has been recent significant expansion in available pharmacotherapy for the management of diabetes and CKD with cardio‐renal benefits, over and above their glycaemic‐lowering benefits.^4^, ^5^ Recent evidence from clinical trials has been incorporated into the Kidney Disease: Improving Global Outcomes (KDIGO) 2022 Clinical Practice Guideline for Diabetes Management in Chronic Kidney Disease, updated after the initial guideline publication in 2020.^6^ This Caring for Australians and New Zealanders with kidney Impairment (CARI) Guidelines commentary summarises the key recommendations contextualised for patients and clinicians in Australia and New Zealand, emphasising the importance of shared decision‐making between clinicians and consumers.

## Guideline summary

The KDIGO 2022 Clinical Practice Guideline for Diabetes Management in Chronic Kidney Disease covers recommendations specific to patients with diabetes and CKD across all stages of CKD, including dialysis and transplant.^6^ However, it does not cover the entity of new‐onset diabetes after transplant and defers pharmacological glycaemic management of type 1 diabetes to diabetes‐specific guidelines. There are 13 graded recommendations based on systematic reviews provided by the Evidence Review Team from Cochrane Kidney and Transplant. In addition, there are 52 practice points supporting the graded recommendations, which are reflective of the expert judgement of the working group. The working group included two consumer representatives, and each graded recommendation includes a domain on the values and preferences of patients. According to our assessment using the Appraisal of Guidelines for Research and Evaluation (AGREE II) instrument, the guidelines have been developed to a high methodological standard (Table 1). Although the working group did include people with lived experience as valued members, a systematic literature review of patient preferences was not undertaken. Additionally, while there was some consideration of the implementation of medication recommendations, the broader implementation across jurisdictions and healthcare settings needs ongoing consideration.

**Table 1:** Guidelines assessment according to the Appraisal of Guidelines for Research and Evaluation (AGREE II) instrument

Guideline	Domain scores (%)						
	Scope and purpose	Stakeholder involvement	Rigour of development	Clarity of presentation	Applicability	Editorial independence	Overall assessment
KDIGO CKD and Diabetes guideline, 2022	100%	71%	88%	100%	79%	64%	86%

The guideline covers key areas including comprehensive diabetes care, glycaemic monitoring and targets, lifestyle measures, glucose‐lowering therapy and care models for managing patients with diabetes and CKD. Highlights in the 2022 guideline are the inclusion of sodium‐glucose co‐transporter 2 (SGLT2) inhibitors and non‐steroidal mineralocorticoid receptor antagonists (nsMRA) into the comprehensive diabetes care chapter, reflecting the evidence for the cardio‐renal benefits of these medicines.^4^, ^7^

## Commentary

### Comprehensive diabetes care

There is a focus on pharmacotherapy with cardio‐renal benefit with graded recommendations for renin‐angiotensin system blockade, SGLT2 inhibitor and nsMRA, in addition to comprehensive cardiovascular disease risk and lifestyle management. Renin‐angiotensin system blockade continues to be the foundation of care for patients with hypertension and albuminuria^8^ (grade 1B recommendation), with a practice point recommending consideration in patients with albuminuria and normal blood pressure.

The 2022 guideline includes a 1A recommendation for SGLT2 inhibitors for use in patients with CKD and estimated glomerular filtration rate (eGFR) *≥*20 mL/min/1.73 m^2^, with evidence from multiple randomised control trials (RCTs) demonstrating improved renal and cardiovascular outcomes compared to placebo.^4^ It is important to note, however, that the renal outcome trials largely recruited patients with albuminuria.^9^, ^10^ The EMPA‐Kidney results were published after this guideline and found no difference in the primary outcome of a composite of progression of kidney disease or death from cardiovascular causes in patients without albuminuria, noting the limitation that this finding was underpowered with a small subgroup and low event numbers.^11^ Recently a meta‐analysis of SGLT2 inhibitor trials found a reduction in progressive CKD across all eGFR and urine albumin‐to‐creatinine ratio subgroups, including the subgroup with normoalbuminuria.^12^ Additionally, SGLT2 inhibitor cardiovascular trials have also shown cardiovascular benefit in patients with diabetes across a range of kidney inclusion criteria.^4^ Graded recommendations based on CKD risk are provided by CARI Guidelines with a strong recommendation for patients with diabetes and CKD.^13^ The Australian Pharmaceutical Benefits Scheme (PBS)^14^, ^15^ and New Zealand Pharmaceutical Schedule^16^ eligibility criteria (Table 2) are more restrictive compared to guidelines including KDIGO^6^ and CARI Guidelines,^13^ as well as drug listing on Therapeutic Goods Australia^17^ and Medsafe,^18^ but largely align with renal outcome trial inclusion criteria. Additionally, in both Australia and New Zealand, there is recognition of the increased cardiovascular risk experienced by Aboriginal, Torres Strait Islander, Māori and Pacific people, with expanded criteria for the prescription of SGLT2 inhibitors to Aboriginal, Torres Strait Islander, Māori and Pacific people with T2D. For example, SGLT2 inhibitors may be prescribed to Aboriginal and Torres Strait Islander peoples in Australia regardless of haemoglobin A1c (HbA1c) and to Māori and Pacific people in New Zealand with HbA1c >53 mmol/mol despite 3 months of an alternate agent.

**Table 2:** Australian Pharmaceutical Benefit Scheme and New Zealand Pharmaceutical Schedule type 2 diabetes and chronic kidney disease indications for SGLT2 inhibitors, nsMRA and GLP1‐RA as of December 2025

SGLT2 inhibitors	
Australian Pharmaceutical Benefit Scheme^14^, ^15^	CKD indication: eGFR ≥20 to <45 mL/min/1.73 m^2^ or eGFR >45 to 90 mL/min/1.73 m^2^ with UACR ≥22.6 mg/mmol, and the patient must be stabilised for at least 4 weeks on either (i) an ACE inhibitor or (ii) an angiotensin II receptor antagonist (unless medically contraindicated) Diabetes indication: T2D in combination with metformin (unless contraindicated/intolerant) regardless of HbA1c in patients at high cardiovascular risk† or T2D in combination with at least one of metformin, sulfonylurea or insulin with inadequate response (HbA1c >7%).
New Zealand Pharmaceutical Schedule^16^	Diabetes indication: T2D not achieving HbA1c target (≤53mmol/mol) despite regular use of at least one blood‐glucose lowering agent for at least 3 months of alternate agents and at high cardiovascular‡ or kidney risk§.

nsMRA	
Australian Pharmaceutical Benefit Scheme^19^	CKD with T2D and an eGFR ≥25 mL/min/1.73 m^2^ and albuminuria ≥22.6 mg/mmol. Patients must be stabilised for at least 4 weeks on an (i) an ACE inhibitor or (ii) an angiotensin II receptor antagonist unless medical contraindicated, AND the treatment must be in combination with an SGLT2 inhibitor (unless contraindicated or intolerant)
New Zealand Pharmaceutical Schedule^16^	Not currently subsidised

GLP1‐RA	
Australian Pharmaceutical Benefit Scheme^14^	Treatment must be used in combination with at least one of metformin, a sulfonylurea or insulin and must be inadequately responsive to at least one of metformin, a sulfonylurea or insulin, and the patient must not have achieved a clinically meaningful glycaemic response with an SGLT2 inhibitor (or contraindicated or intolerant requiring discontinuation) Not to be prescribed in combination with an SLGT2 inhibitor, unless SGLT2 inhibitor is prescribed for a non‐diabetes indication
New Zealand Pharmaceutical Schedule^16^	Diabetes indication: T2D not achieving HbA1c target (≤53 mmol/mol) despite regular use of all of the following blood‐glucose lowering agents for at least 6 months, where clinically appropriate: empagliflozin, metformin and vildagliptin and at high cardiovascular‡ or kidney risk§ Not to be prescribed in combination with an SLGT2 inhibitor, unless SGLT2 inhibitor is prescribed for heart failure indication

† High CVD risk AUS PBS defined as: established CVD, estimated CVD risk of ≥10% over 5 years, or identifies as Aboriginal or Torres Strait Islander.

‡ High CVD risk NZ pharmaceutical schedule defined as: Māori or Pacific ethnicity, pre‐existing cardiovascular risk or risk equivalent, absolute 5‐year cardiovascular disease risk of 15% or greater, high lifetime cardiovascular risk due to being diagnosed with T2D during childhood or as a young adult.

§ Kidney risk NZ pharmaceutical schedule defined as: diabetic kidney disease defined as persistent UACR ≥3 mg/mmol and/or eGFR ≤60 mL/min/1.73 m^2^.

Abbreviations: ACE, angiotensin‐converting enzyme; eGFR, estimated glomerular filtration rate; SGLT2, sodium‐glucose co‐transporter 2; UACR, urine albumin‐to‐creatinine ratio.

There is a new graded recommendation for the use of nsMRA in patients with eGFR ≥25 mL/min/1.73 m^2^, with normal serum potassium and albuminuria (≥3 mg/mmol) despite the use of a RAS inhibitor. The guideline acknowledges that there is a paucity of data on the benefits of concomitant nsMRA and SGLT2 inhibitor use. Secondly, there is a need for monitoring of potassium and potential cost implications. In the Australian context, nsMRA are available on the PBS but are limited to patients with CKD with eGFR ≥25 mL/min/1.73 m^2^, type 2 diabetes (T2D) and albuminuria ≥22.6 mg/mmol, despite the use of a RAS inhibitor and SGLT2 inhibitor (Table 2).^19^ nsMRA are currently unavailable on the New Zealand subsidised Pharmaceutical Schedule. Steroidal mineralocorticoid receptor antagonists may be considered an alternative to nsMRA and are widely available at low cost; however, in the absence of kidney outcome data from large‐scale RCTs, benefits in the reduction of albuminuria^20^ need to be weighed against the risk of hyperkalaemia and acute kidney injury.^6^

Glucagon‐like peptide‐1 receptor agonists (GLP1‐RAs) are not included in the comprehensive care chapter; however, the 2022 guideline preceded relevant kidney outcome trial results, that is, the FLOW study.^21^ GLP1‐RAs are now considered a pillar of therapy^22^ and have been shown to improve cardiovascular and kidney outcomes in patients with T2D over and above weight loss or glucose‐lowering benefits.^5^, ^21^ Currently, GLP1‐RAs are available on the PBS in Australia and on the Pharmaceutical Schedule in New Zealand, with use limited to patients with inadequate diabetes control despite standard therapies.^14^, ^16^ Additionally, GLP1‐RAs cannot be used in combination with an SGLT2 inhibitor unless the SGLT2 inhibitor is used for another indication (Table 2). Consumers have highlighted the importance of improving accessibility and equity of GLP1‐RAs, particularly due to supply shortages and limited qualifying criteria for prescriptions. It is anticipated that further evidence from trials investigating outcomes of combination therapy will likely inform future recommendations regarding combination therapy and, through shared decision‐making between clinicians and consumers, support personalised precision medicine.

### Glycaemic monitoring and targets

The guideline has a 1C recommendation for an individualised HbA1c target between 6.5% and 8% (48–64 mmol/mol). This is consistent with recommendations from other peak bodies,^23^, ^24^ which recommend a general target of 7% (53 mmol/mol). However, in those for whom prevention of complications is the primary goal, a lower target is preferred, and in those with multi‐morbidity, frequent hypoglycaemia or short life expectancy, a higher target may be preferred. HbA1c remains the recommended test for monitoring diabetes, notwithstanding the limitation of HbA1c in advanced CKD.

### Lifestyle measures

There have been no changes to the lifestyle recommendations from the 2020 guideline, with recommendations largely derived from large studies of the general population. Key graded recommendations include sodium reduction to <2 g sodium per day and moderate‐intensity physical activity for at least 150 min per week. These recommendations are consistent with peak body recommendations from the heart, kidney and diabetes foundations.^25^, ^26^, ^27^, ^28^, ^29^, ^30^ It is noted that no graded recommendations are provided for weight management; however, the chapter on comprehensive diabetes care includes healthy diet, physical activity, smoking cessation and weight management as part of comprehensive lifestyle management. These recommendations are highly valuable to consumers, as the inclusion of general recommendations for health and well‐being promotes a sense of agency and empowerment for people living with CKD and diabetes.

### Glucose‐lowering therapies

Recommendations for glucose‐lowering therapies have undergone a significant update in the 2022 guideline, with priority given to agents with additional cardio‐renal benefits. The standard of care is metformin (dose modify in eGFR <30 mL/min/1.73 m^2^ and discontinue in patients with eGFR <15 mL/min/1.73 m^2^,^31^) and SGLT2 inhibitors, with add‐on therapies being GLP1‐RA (preferred), followed by alternate agents. There is a welcome focus on precision medicine in choice of agent, taking into consideration patient comorbidities and preference through shared decision‐making between caregivers and consumers, as well as medication cost and availability.

### Approaches to management including care models

The final chapter of the guideline considers the structural and policy aspects of providing comprehensive diabetes and kidney care. The importance of self‐management educational programmes (1C recommendation) and integrated care approaches are highlighted. Despite the grade 2C evidence for integrated care models, the working group acknowledges that the complex care needs of patients with diabetes and CKD can only be met through a team‐based approach. This is consistent with prior Australian research describing challenges of fragmented care.^32^ It is also essential to ensure that self‐management programmes and care models are adequately resourced and designed with consumers to ensure cultural sensitivity and accessibility.

## Future directions and conclusions

The 2022 KDIGO clinical practice guideline for the management of diabetes and CKD provides a comprehensive guideline for the management of diabetes and CKD, with an important focus on new pharmacotherapy with cardio‐renal benefit. It is essential that our health policy and systems, in addition to clinicians, work to ensure implementation of therapies with a strong evidence base for improving outcomes for people with diabetes and CKD.

## Data Availability

Data sharing not applicable to this article as no datasets were generated or analysed during the current study.

## Appendix I

### CARI Guidelines Steering Committee investigators

Emily See (Melbourne Medical School, The University of Melbourne, Melbourne, Vic., Australia; Department of Intensive Care, Austin Health, Heidelberg, Vic., Australia), Leanne Brown (School of Nursing and Institute of Health and Biomedical Innovation, Queensland University of Technology, Brisbane, Qld, Australia; School of Nursing and Midwifery, Griffith University, Brisbane, Qld, Australia; Murtupuni, Centre for Rural and Remote Health, James Cook University, Cairns, Qld, Australia), Helen Coolican (Consumer Partner, Australia), Jonathan Craig (College of Medicine and Health, Flinders University, Adelaide, SA, Australia), Vanessa Cullen (Consumer Partner, Australia), Jeffrey Ha (George Institute of Global Health. UNSW, Sydney, Australia; Department of Renal Medicine, St George Hospital, Sydney, Australia), Rathika Krishnasamy (Faculty of Medicine, University of Queensland, Brisbane, Qld, Australia; Sunshine Coast University Hospital, Birtinya, Qld, Australia), Kelly Lambert (Graduate School of Medicine, University of Wollongong, Wollongong, NSW, Australia), Casey Light (School of Nursing, Curtin University, Perth, WA, Australia; Armadale Hospital, Mount Nasura, WA, Australia), Emmy O'Neill (Consumer Partner, Australia; Transplant Australia, Brisbane, Qld, Australia), Hannah Smith (Consumer Partner, Australia), Andrea Viecelli (Faculty of Medicine, University of Queensland, Brisbane, Qld, Australia).
